# Entrustable Professional Activities for Pharmacists Carrying Out Medication Management Reviews

**DOI:** 10.3390/pharmacy14030075

**Published:** 2026-05-19

**Authors:** Carmen Abeyaratne, Nadine Fawal, Kirsten Galbraith

**Affiliations:** Faculty of Pharmacy and Pharmaceutical Sciences, Monash University, Melbourne, VIC 3052, Australia; nfaw0001@gmail.com (N.F.); kirstie.galbraith@monash.edu (K.G.)

**Keywords:** Entrustable Professional Activities, EPAs, pharmacy, medication management reviews

## Abstract

This study aimed to evaluate the suitability of previously validated Entrustable Professional Activities (EPAs) for pharmacists carrying out medication management reviews (MMRs) and further align them with nationally accepted performance outcomes (POs). This was a two-phase study involving Subject-Matter Experts (SMEs). Phase 1 aimed at assessing the relevance of 14 EPAs previously validated for use in a provisionally registered pharmacist training programme to the role of MMR pharmacists utilising a five-point Likert scale. A 70% threshold for “agree” and “strongly agree” was used to qualify an EPA for phase 2. During phase 2, EPAs were mapped for their ability to demonstrate APC performance outcomes for MMRs and the results were descriptively reported. Of the eight SMEs recruited, seven completed both surveys. Of the 14 EPAs, 12 qualified for phase 2. In phase 2, the 12 EPAs were mapped against 24 POs. Five EPAs were matched with 100% agreement to six POs. To our knowledge, this is the first study of its kind to map pre-validated EPAs to POs for credentialing in the scope of MMR pharmacists in Australia. A total of 12 EPAs were applicable to pharmacists working in the MMR space, and five were able to be directly mapped to six POs.

## 1. Introduction

In Australia, pharmacists have been eligible to undertake extra training to provide remunerated medication management review (MMR) services (including Residential Medication Management Reviews (RMMR) in residential aged care settings) for over 20 years [1]. MMRs are conducted to ensure the safety and effectiveness of medication(s) and prevent any medication-related problems while considering patient status and comorbidities [2]. Credentialled MMR pharmacists play an integral role in reviewing complex medication regimes, identifying causes of medication-related problems, providing solutions to such problems, and identifying gaps in care [3]. Previous studies investigating RMMRs have shown that medication reviews by credentialled pharmacists identified a mean of 2.7–3.9 medication-related problems per patient [4]. Furthermore, a systematic review identified that 43% of aged care residents use one or more inappropriate medications [5]. While these funded services have been available for many years, policy changes have hindered programme expansion. In 2018–2019 it was reported that home medication review availability was 3–6 times less than the population need, while the number of pharmacists completing medication reviews had stabilised since 2014 [6]. To address these gaps, Sluggett et al. recommended pharmacists and governing bodies provide “extra services” and ensure that “workforce training and education” is provided [6].

In 2021, The Royal Commission into Aged Care Quality and Safety (The Royal Commission) identified residential aged care as an essential area of improvement and called for “fundamental reform of the aged care system” [7]. The final report included 148 recommendations, including the need “to provide comprehensive medication management reviews by pharmacists to prevent harmful medicine interactions, overuse of medication or chemical restraint via inappropriate use of antipsychotics” [7]. In 2023, the Australian Department of Health and Aged Care requested the Australian Pharmacy Council (APC), the organisation responsible for advancing and assuring quality in pharmacy education, develop new accreditation standards and an accreditation system for education programmes to accredit pharmacists (as an additional post-graduate qualification) to work as Aged-Care On-site Pharmacists (ACOPs) and conduct MMRs [8]. This was achieved by following the APC’s well established, consultative approach to the development of standards, and resulted in a set of Accreditation Standards, accompanying performance outcomes (POs), and a system to accredit education providers [8]. The use of POs in the accreditation approach seeks to ensure that education programmes provide a systematic approach to credentialling pharmacists that demonstrate performance for these roles at the required levels [8].

The concept of Entrustable Professional Activities (EPAs), originally developed by Ten Cate, has become widely used in health care for competency-based training and assessments of clinical activities [9,10]. The overall aim of EPAs is to allow for more targeted evaluations of “knowledge, skills, and attitudes with a recognised output of professional labour, independently executable within a time frame, observable and measurable in its process and outcome” [10]. Previous work undertaken by the APC relating to provisionally registered (intern) pharmacists resulted in a blueprint mapping of assessment types to POs [11]. One such assessment type recommended was EPAs.

To ensure EPAs are relevant and have the ability to be assessed in the area of interest they should, ideally, be pre-validated with the QUEPA (Quality of EPA) tool or EQual (Queen’s EPA quality) rubric [12]. In 2021, Abeyaratne et al. developed and validated 14 EPAs using the EQual rubric for use in the workplace by provisionally registered pharmacists [13]. These EPAs were found to be feasible and appropriate workplace-based assessment tools for intern pharmacists working in the hospital and community setting [14]. EPAs that were previously validated using the recommended tools by Ten Cate [15] were chosen for this study to ensure quality and robust EPAs. Furthermore, although the EPAs were designed for provisionally registered pharmacists, the EPA title and description is an activity expected for a registered pharmacist, including those undertaking MMRs. This study did not evaluate the entrustment level of the learner in regard to their ability to carry out the EPA.

EPAs in other pharmacy practice specialities, including hospice and palliative care, have been developed [16]. To our knowledge, no validated EPAs focused on credentialled pharmacists working in the speciality area of MMR in Australia have been developed. This research aims to evaluate whether EPAs developed and validated for use in a provisionally registered training programme for pharmacists are relevant for use in a program for pharmacists carrying out MMRs and align with POs from the APC for MMR pharmacists.

## 2. Materials and Methods

### 2.1. Study Design

The study was a prospective quantitative survey design with two sequential phases, each of which involved a survey hosted on the Qualtrics platform. The surveys were delivered during April and May 2024.

### 2.2. Study Subjects

Participants were Subject-Matter Experts (SMEs) with over 10 years of experience specialising in conducting MMRs and/or working as an ACOP. All SMEs had a recognised higher qualification to carry out MMRs and were chosen based on their high level of knowledge in this area. Participants were from academic, community, hospital and professional peak body settings. They were recruited via existing professional relationships with the authors and referrals from participants already recruited. Potential participants were also identified through a local Home Medicines Review database. Informed consent was obtained.

### 2.3. Phase 1

The phase 1 survey focused on assessing the relevance of 14 EPAs, previously validated for use with provisionally registered pharmacists, [13] to the role of MMR pharmacists. SMEs were asked to rate the relevance of each EPA and description to the work of performing MMRs, using a five-point Likert scale ranging from strongly disagree to strongly agree. For EPAs where either strongly disagree or disagree was selected, SMEs were encouraged to provide additional comments as to why the task was not relevant for the role of an MMR pharmacist. The survey link was circulated via email and data was exported to an Excel spreadsheet for analysis. The survey was open for seven days, with an email reminder sent after five days. A threshold of 70% combined values for “agree” and “strongly agree” was chosen for an EPA to be eligible to be included in phase 2, which aligned with a previous study [17]. The additional comments provided by SMEs were not extensive enough to warrant any further analysis.

### 2.4. Phase 2

The phase 2 survey required SMEs to map the relevant EPAs and descriptions from phase 1 to the 24 APC POs for pharmacists conducting MMRs [8]. SMEs were presented with each PO individually and asked to select which EPAs could be used to demonstrate the relevant performance indicator. Multiple EPAs could be selected for each PO. Only EPA tasks that met the 70% threshold score consisting of agree/strongly agree from phase 1 were included in phase 2. The phase 2 survey was open for seven days, with an emailed reminder after five days. Data from the phase 2 survey were reported descriptively using numbers, which represented the number of SMEs that selected an EPA that can be used to assess a PO. A threshold of 100% agreement for a PO aligning with an EPA was chosen for phase 2. Any POs that had an EPA that reflected total agreement among SMEs, a value of 7, were extracted and represented separately in a data table. A 100% consensus measure between participants was chosen in this phase as this was a pilot study with a focus on establishing the feasibility of specific EPAs for pharmacists carrying out MMRs.

## 3. Results

### 3.1. Phase 1

Eight SMEs were recruited, of which seven completed the survey by the deadline. 12 of the 14 EPAs met the criteria for inclusion in phase 2 (Table 1). The two EPAs that did not proceed into phase 2 were: “Dispenses Medicines” (agree/strongly agree 14.3%, *n* = 1) and “Prepares and Dispenses Extemporaneous Products” (agree/strongly agree 0%). Additional comments regarding these two EPAs are provided in Table 2.

### 3.2. Phase 2

The seven SMEs from phase 1 all participated in this phase; however, only four responded to all questions (one SME responded to 18 out of 24 questions and two SMEs responded to 19 out of 24 questions) (Appendix A). Although these responses were incomplete, they were still included in the analysis. There was 100% agreement that five EPAs could be used to demonstrate six performance outcomes (Table 3).

## 4. Discussion

The suitability and relevance of pre-validated EPAs from a training programme for provisionally registered pharmacists were evaluated for their use in credentialling pharmacists carrying out MMRs. Currently, there is limited research that has focused primarily on validated EPAs tailored for pharmacists conducting MMRs [18].

Our phase 1 survey tested the relevance of pre-validated EPAs for pharmacists carrying out MMRs. A total of 12 EPAs were deemed relevant (reached an agreement of more than 70%) by seven SMEs (Table 1). Phase 1 had an expected outcome, with “Dispenses Medicines” and “Prepares and Dispenses Extemporaneous Products” excluded as they did not reflect the typical daily duties of MMR pharmacists, according to the SMEs and our prior understanding of the role of an MMR pharmacist [2,3,19]. SMEs noted that although MMR pharmacists may not dispense medication or prepare extemporaneous products, they “need to understand the processes and structures involved” as this understanding may be “beneficial for review pharmacists”. In saying so, “demonstration of the tasks are not required” (Table 2). These findings may require further investigation as to the role dispensing medication and preparing extemporaneous products play in regard to MMR pharmacists. Furthermore, awareness and understanding of these tasks are still required to carry out MMRs. It is also important to acknowledge that “Dispenses Medicines” and “Prepares and Dispenses Extemporaneous Products” are entry-level requirements for all pharmacists, but are not specific to this expanded MMR role.

In phase 2, many of the EPAs were not consistently mapped to the POs. This inconsistency suggests that not all POs can be assessed within the MMR role. Furthermore, it highlights how some EPAs may need further refinement and screening to better describe the tasks of a pharmacist carrying out MMRs. Furthermore, low survey completion rate in phase 2 also contributed to this inconsistency. Out of the 24 POs, six were able to be demonstrated by five EPAs. These results indicate that the five validated EPAs from our phase 1 survey can reliably be used to demonstrate performance in undertaking MMRs as all SMEs were able to map them to the POs listed by the APC, which is essential in ascertaining their relevance to the context of credentialling specialty roles such as MMR pharmacists. EPAs are one example of the many tools available to demonstrate candidates meeting specified POs and not all performance indicators can be captured using EPAs as an assessment [20].

Our results are comparable with those of Villa et al. who identified and evaluated a set of EPAs relevant to emergency medicine fellowship [17]. Villa et al. focused on defining and implementing EPAs as a component of their competency-based training approach, which aligns with our study regarding pharmacists carrying out MMRs [17]. Both studies aimed to identify EPAs that are relevant to training certain health care professionals. In line with Villa et al., we also engaged with experts in the field to evaluate each EPA, to ensure applicability to real-world settings [17]. Furthermore, we adopted a 70% agreement threshold in our methodology based on the work of Villa et al. to determine a formal cutoff for the data and standardise the approach to data analysis [17].

The significant difference between our study and previous research is our focus on mapping each EPA to POs. The study by Villa et al. demonstrated the relevance of EPAs to the role of medical students and gathered significant agreement amongst their SMEs [17]. Our study took the additional step of ensuring that each relevant EPA from phase 1 also matched to the POs set by the APC. This additional step provides a more detailed evaluation of EPAs for pharmacists undergoing MMR training. Furthermore, EPAs used in this study were more narrowly focused on pharmacy as they were extracted from an Intern Pharmacist Program. Our approach to employ EPAs from a particular educational setting guarantees that the EPAs are applicable and relevant to the targeted training programmes. Another difference compared to Villa et al. was that the SMEs were only presented with the EPA title but not the description, which may have caused ambiguity [17]. Our study presented the SMEs with the EPA title and description to ensure a thorough understanding of the EPA task.

### 4.1. Limitations

Despite our findings, our study had several limitations. Firstly, the sample size of only seven respondents limits the generalisability and validity of the findings. This was a pilot exploratory study to evaluate whether EPAs developed and validated for use in a provisionally registered training programme for pharmacists were relevant for use in a program for pharmacists carrying out MMRs. A larger sample size as used in other studies [17,21] could provide more robust results applicable to the broader MMR pharmacist population. We utilised pre-validated EPAs from a previous study [13]; if we developed a new set of EPAs there could be other applicable EPAs for pharmacists carrying out MMRs. Additionally, this study explores EPAs in the Australian MMR context, limiting transferability to MMR programmes in other regions.

Survey fatigue could also be a limitation due to the high volume of questions in the phase 2 survey [22]. Although respondents could save their progress and return later on any device or internet network of their choosing, this may have led to some incomplete questions. Lastly, the absence of a “none of the above” option during phase 2 may have led some SMEs to have left blank responses with questions either unanswered or skipped. This contributed to unclear representations of their experiences, affecting the reliability and validity of the results. However, including all the incomplete responses from phase 2 ensured that the results were not biased and were a true reflection of the participants’ views. The phase 1 survey only asked for additional comments when respondents selected disagree or strongly disagree. This may have influenced EPAs that were barely over the identified threshold of 70%. These limitations highlight the need for further research with larger sample sizes, more comprehensive EPA sets, and improved survey design to enhance the validity and reliability of the findings.

### 4.2. Future Directions

The results contribute to the existing gap by demonstrating which pre-validated EPAs are applicable to the observable tasks performed by MMR pharmacists in Australia that align with the POs from the APC. Moving forward a trial/pilot could potentially be established in which pharmacists who wish to be accredited to carry out MMRs and their supervisors can use the EPAs in order to assess the knowledge, skills, and capabilities of the learner. Additionally, the use of EPAs may not be limited only to MMRs, it can also extend to the role of an Aged-Care On-site Pharmacist or other areas of specialty/scope of practice.

## 5. Conclusions

Five EPAs previously validated for use in a provisionally registered pharmacy training programme were deemed by SMEs to be suitable for demonstrating six MMR performance outcomes. The positive findings encourage further exploration and implementation of EPAs to improve the quality of care provided by pharmacists and ensure their alignment with professional standards and expectations.

## Figures and Tables

**Table 1:** Overall responses by SMEs to the phase 1 survey in assessing the relevance of 14 pre-validated EPAs for pharmacists carrying out MMRs.

EPAs	Likert Scale Response (*n* = 7)					“Agree” and “Strongly Agree” Combined
Entrustable Professional Activities [13]	Strongly Disagree	Disagree	Neutral	Agree	Strongly Agree	Strongly Agree/Agree *n* (%)
Documents and records relevant information	0	0	0	0	7	7 (100%)
Dispenses medicines	4	1	1	1	0	1 (14.3%)
Prepares and dispenses extemporaneous products	4	3	0	0	0	0 (0%)
Provides appropriate patient counselling	0	0	0	2	5	7 (100%)
Obtains best possible medication and patient history	0	0	0	0	7	7 (100%)
Makes appropriate and effective clinical decisions	0	0	0	0	7	7 (100%)
Provides primary health care	0	0	2	3	2	5 (71.4%)
Assesses health and medication histories	0	0	0	0	7	7 (100%)
Performs medication reviews	0	0	0	1	6	7 (100%)
Formulates patient-centred management plans	0	0	0	1	6	7 (100%)
Provides person-centred care	0	0	0	1	6	7 (100%)
Applies the principles of Quality Use of Medicines	0	0	0	0	7	7 (100%)
Enhances adherence, safe and effective administration of medications	0	0	0	0	7	7 (100%)
Practises harm minimisation	0	0	2	3	2	5 (71.4%)

**Table 2:** Written comments from SMEs about why certain EPAs are not relevant for pharmacists carrying out MMRs.

Entrustable Professional Activities (EPAs)	Comments from Subject-Matter Experts (SMEs)
**Dispenses medicines** Demonstrates ability to consistently dispense medicines safely and accurately in accordance with current legislation, scope of practice, PharmBA Guidelines.	“Dispensing is not part of MMR’s. Understanding the dispensing history and what information it can yield is, however, a summary of recommendations from an MMR should ideally be added to the comments field in the dispensing history. I am a non-dispensing, consultant pharmacist.” “Not part of the role” “Dispensing is not a role for MMR pharmacist, MMRs rarely occur in a setting where dispensing occurs. (i.e., not in community pharmacy, more likely in persons home or Residential Aged Care Facility)” “MMR pharmacists can be (and often are) non-dispensing pharmacists.” “An MMR pharmacist needs to understand dispensing, the processes and structures involved (e.g., Pharmaceutical Benefits Scheme, Escripts, active script list, repeats etc) but does not need to demonstrate dispensing medicines.”
**Prepares and dispenses extemporaneous products** Demonstrates ability to extemporaneously prepare and supply compounded medications safely and accurately.	“For an MMR Pharmacist it is understanding what is in the extemp and how that may impact the patient/interact with other medication is what is relevant to the role. Needless to say both dispensing and extemp are still core skills in basic (but not advanced) pharmacy practice.” “Not part of the role—separate to the supply pharmacy” “This is not a task that I think would ever be expected of a pharmacist completing an HMR or RMMR. An understanding of how these products are sourced by patients who require them (e.g., topical products for particular conditions) may be beneficial for review pharmacists, however the preparation and dispensing of them is not relevant to the medication review process.” “Skill sets needed to adequately & competently do either task are quite different” “Not a dispensing role nor a role which involves preparing products” “MMR pharmacists do not need to be able to compound medications.” “An MMR pharmacist needs to know about compounding pharmacies and accessing compounded medicines but does not need to demonstrate compounding.”

**Table 3:** MMR performance outcomes and EPA mapping with 100% SME (*n* = 7) agreement.

Performance Outcomes (POs)	Entrustable Professional Activities (EPAs)
1.1 Engages with consumers in a respectful, inclusive and culturally appropriate manner (a) Promotes, maintains and advocates for cultural safety, respect and responsiveness particularly in relation to Aboriginal and Torres Strait Islander peoples (b) Recognises unique issues, challenges and health care needs of vulnerable populations, including people who identify as LGBTQIA+, or those living with a disability, and delivers care appropriate to the consumer’s needs and goals	**Provides person-centred care:** Demonstrates ability to implement and deliver socially accountable person-centred care, encompassing patients’ rights, values and beliefs. Maintains privacy and confidentiality and ensures informed consent is obtained.
2.4 Produces clear and concise clinical documentation using standardised communication tools and according to best-practice guidelines relevant for the practice setting (a) Produces clear and concise MMR reports using standardised communication tools and according to best-practice guidelines	**Documents and records relevant information:** Demonstrates ability to document, communicate and record relevant information, findings, decisions, recommendations and other information accurately, concisely and in a timely manner, taking into account privacy and confidentiality.
2.5 Documents, communicates and records clinical reasoning, actions and recommendations ensuring the privacy and confidentiality of information is maintained (a) Makes and shares appropriate records with the consumer and health professionals including uploading to a clinical documentation system or consumer health record where digitally enabled	**Documents and records relevant information:** Demonstrates ability to document, communicate and record relevant information, findings, decisions, recommendations and other information accurately, concisely and in a timely manner, taking into account privacy and confidentiality.
3.1 Obtains and documents an accurate Best Possible Medication History (BPMH) for a consumer using relevant sources of health information (a) Collects, collates and considers available and relevant health, medical and medication information from the referral, referring practitioner, community pharmacy/ies, other health professionals, consumer and carers	**Obtains best possible medication and patient history:** Demonstrates ability to obtain relevant health, medical and an accurate medication history using a comprehensive, standardised approach.
3.3 Develops evidence-based and person-centred recommendations to address identified problems and optimise consumer care (a) Considers the consumer’s treatment goals (including advance care directives) when making recommendations	**Makes appropriate and effective clinical decisions:** Demonstrates ability to implement appropriate and effective actions and recommendations which support safe, rational and cost-effective use of medicines and other health care options.
3.8 Assesses minor conditions and provides management recommendations, within scope of practice, including pharmacological, non-pharmacological and referral options (Note: If identified during MMR)	**Provides primary health care:** Assesses signs, symptoms and manages primary health conditions within the scope of pharmacy practice. Where appropriate provides pharmacological and non-pharmacological treatments and/or referral to other health professionals when appropriate

## Data Availability

The original contributions presented in this study are included in the article. Further inquiries can be directed to the corresponding author.

## Appendix A

**Table A1:** SME responses to APC medication management review performance outcomes mapped to EPAs from phase 1.

Medication Management Review Pharmacist Performance Outcome	EPAs											
	Documents and Records Relevant Information	Provides Appropriate Patient Counselling	Obtains Best Possible Medication and Patient History	Makes Appropriate and Effective Clinical Decisions	Provides Primary Health Care	Assesses Health and Medication Histories	Performs Medication Reviews	Formulates Patient-Centred Management Plans	Provides Person-Centred Care	Applies the Principles of Quality Use of Medicines	Enhances Adherence, Safe and Effective Administration of Medications	Practises Harm Minimisation
SMEs’ Responses (*n* = 7)												
1.1 Engages with consumers in a respectful, inclusive and culturally appropriate manner	1	6	4	3	4	3	1	4	7	1	2	2
1.2 Provides and ensures understanding of relevant information to enable the consumer or substitute decision-maker to give informed consent	5	3	0	1	0	0	0	0	6	0	0	0
1.3 Involves the consumer in shared decision-making about their care	2	4	1	1	2	0	1	4	6	1	1	0
1.4 Maintains the privacy and confidentiality of the consumers personal information through safe and secure communication and storage mechanisms	5	0	1	0	0	1	0	0	4	0	0	0
1.5 Complies with relevant legislative, regulatory and professional codes, guidelines and standards	4	1	2	3	0	1	4	1	2	4	1	1
2.1 Communicates in a culturally safe manner all medicine-related information to consumers, carers and families in a form they can use and understand, facilitates shared decision-making, advocacy and self-determination	0	5	2	0	1	0	0	2	6	0	1	0
2.2 Recognises and respects the roles of consumers, carers and their families, other care providers and health professionals and establishes effective working relationships	3	3	2	2	4	2	1	5	4	2	3	1
2.3 Collaborates and communicates clearly and effectively with other health professionals and care providers to conduct comprehensive and person-centred medication reviews, multidisciplinary meetings, or case conferences	5	0	2	4	2	4	4	5	3	4	1	1
2.4 Produces clear and concise clinical documentation using standardised communication tools and according to best practice guidelines relevant for the practice setting	7	1	2	2	1	2	6	2	1	4	1	1
2.5 Documents, communicates and records clinical reasoning, actions and recommendations ensuring the privacy and confidentiality of information is maintained	7	0	1	3	0	2	1	3	1	3	1	1
3.1 Obtains and documents an accurate Best Possible Medication History (BPMH) for a consumer using relevant sources of health information	5	0	7	1	0	4	2	1	0	0	0	0
3.2 Conducts a comprehensive medication management review to identify potential or actual medicine-related problems	3	1	4	3	1	3	6	3	3	2	3	0
3.3 Develops evidence-based and person-centred recommendations to address identified problems and optimise consumers’ care	2	3	1	7	1	3	3	4	3	5	3	1
3.4 Collaborates with the consumer, prescribers and other health professionals and care providers to develop and action an agreed medication management plan	3	1	2	3	1	1	3	6	2	3	1	1
3.5 Answers medicine-related queries and provides medicine information and advice to consumers, other health professionals and care providers accurately, appropriately, and safely	2	6	1	4	4	0	0	0	0	5	3	2
3.6 Provides information and tools to support consumers to monitor their health conditions and manage their medicines in a safe and effective way to prevent complications and maintain independence as much as possible	1	6	1	2	2	1	0	4	4	4	6	2
3.7 Develops and recommends a plan to monitor the consumer’s response to medication management plans in relation to safety and efficacy	2	1	1	1	0	0	1	6	1	2	3	0
3.8 Assesses minor conditions and provides management recommendations, within scope of practice, including pharmacological, non-pharmacological and referral options (Note: If identified during MMR)	2	4	1	2	7	0	0	0	1	2	0	1
3.9 Administers medicines (including injectable formulations) in accordance with current jurisdiction-specific legislation, organisational policies, scope of practice, and PharmBA Guidelines, if required (Note: If required to assist consumer)	2	1	0	0	2	1	0	0	0	0	2	0
4.1 Applies clinical governance principles to professional practice, including a commitment to continuous quality improvement	2	0	0	1	0	0	2	1	0	4	2	1
4.2 Proactively identifies, plans, prioritises and evaluates quality improvement activities	2	0	0	0	0	0	0	0	0	3	1	0
4.7 Analyses, reports and provides recommendations in response to potential or actual medicine-related incidents and adverse events	5	1	1	1	0	1	1	1	0	2	2	3
4.11 Maintains, complies and regularly reviews policies and processes to ensure evidence-based, safe, and quality provision of health care	3	0	0	0	0	0	0	0	0	5	2	1
4.13 Advises on appropriate and safe use of medicines, including storage and disposal	1	4	0	2	0	1	1	1	0	3	2	5
